# Trigeminal Nerve Asymmetry in Horses With Idiopathic Trigeminal‐Mediated Headshaking: A Retrospective Case‐Control Magnetic Resonance Imaging Study

**DOI:** 10.1111/jvim.70196

**Published:** 2025-07-31

**Authors:** Frederik Heun, Julien Delarocque, Karsten Feige, Maren Hellige

**Affiliations:** ^1^ Clinic for Horses University of Veterinary Medicine Hannover, Foundation Hannover Germany

**Keywords:** brain, equine, magnetic resonance imaging, neuralgia, neuropathy

## Abstract

**Background:**

Nerve atrophy results in trigeminal nerve (TN) asymmetry detectable by magnetic resonance imaging (MRI) in humans, but similar studies have not been performed in horses with idiopathic trigeminal‐mediated headshaking (ITMHS).

**Hypothesis:**

Horses with ITMHS show greater MRI‐detected trigeminal‐nerve asymmetry than controls.

**Animals:**

A total of 20 adult horses with ITMHS and six unaffected control horses.

**Methods:**

Retrospective case‐control study of the TN cross‐sectional area (TNCSA) based on 3‐Tesla MRI scans of the equine brain. TNCSA and its side‐to‐side differences at four defined measurement points were compared within the two study groups using a linear mixed model. Intraclass correlation coefficient analysis was used to evaluate intra‐rater repeatability. The primary outcome was side‐to‐side TNCSA asymmetry, minimizing confounding effects such as body size.

**Results:**

Significantly greater TNCSA side‐to‐side differences (asymmetry of TN) were detected in horses with ITMHS (*F*_(3,70)_ = 11.271, *p* < 0.001). Horses with ITMHS exhibited a 4.1 to 7.6‐fold greater TN asymmetry compared to control horses. Absolute TNCSA did not differ significantly between groups but was influenced by body weight. Measurements demonstrated excellent repeatability, and tentative cut‐off values could be calculated to discriminate between ITMHS and control horses based on TNCSA asymmetry.

**Conclusions and Clinical Importance:**

The asymmetry of the TNCSA in horses with ITMHS indicates unilateral or asymmetric disease of the TN. The present study highlights the value of MRI examinations in ITMHS and could pave the way for targeted therapeutic approaches.

**Abbreviations**
HS: headshaking
ICC: intraclass correlation coefficient
ITMHS: idiopathic trigeminal‐mediated headshaking
mm: millimeter
MP: measurement point
MRI: magnetic resonance imaging
msec: millisecond
TE: echo time
TN: trigeminal nerve
TR: time of repetition

## 1 Introduction

Headshaking (HS) occurs with a prevalence of 1% to 4.6% in the equine population [1, 2], with idiopathic trigeminal‐mediated HS (ITMHS) as the most common diagnosis in affected horses [3]. ITMHS can cause pain and, consequently, reduced quality of life [4]. Typically, adult horses of various breeds, ages, and disciplines are affected, with geldings being overrepresented [1, 2, 5]. Despite extensive research, the etiology of ITMHS remains uncertain [4]. However, there is evidence of trigeminal nerve (TN) dysfunction. Electrophysiological examinations reveal decreased stimulation thresholds [6, 7, 8] associated with ITMHS, suggesting hyperexcitability of the sensory part of the TN. Although preliminary data suggest latency changes [9], larger studies do not confirm this. Electrodiagnostic methods can support the diagnosis by detecting decreased stimulation thresholds [7, 8], but results might be normal in horses not exhibiting clinical signs at the time of testing, reflecting the condition's episodic nature. Diagnosis of ITMHS remains one of exclusion, made after thorough evaluation rules out all identifiable causes of HS, and the remaining clinical picture—including history, signalment, and characteristic HS behavior—strongly supports a trigeminal‐mediated origin [3]. Current treatment options result in inconsistent or short‐term improvement [3, 10].

In human medicine, trigeminal neuralgia is a neuropathic facial pain disorder [11, 12] considered comparable to ITMHS [4, 11]. Trigeminal neuralgia is characterized by predominantly unilateral TN neuropathy, most commonly resulting from arterial compression at the root‐entry zone, leading to neurovascular conflict [11, 13]. Although vascular compression and demyelination are common features, some patients show atrophy of the affected nerve on magnetic resonance imaging (MRI) [14, 15]. However, this finding is not consistent [11], and up to one‐third of patients show no detectable neurovascular conflict [13], suggesting heterogeneous pathophysiology. Although no neurovascular conflict has been identified in horses, similarities between human trigeminal neuralgia and ITMHS are suspected based on facial pain signs. Comprehensive MRI studies in horses with ITMHS are lacking.

The TN is reliably detectable in equine head MRI [16, 17, 18]. The TN root, the trigeminal ganglion, and main nerve branches are distinguishable. The mandibular nerve exits via the oval fossa, while the ophthalmic and maxillary nerves exit via the orbital fissure and the foramen rotundum, respectively [17]. MRI‐based depiction of these branches is described in comparison to anatomical slides [18, 19]. TN diameters are reported [19]. A cadaver study recently describes the subarachnoid space's microarchitecture around the trigeminal ganglion and its anatomical variation [20]. However, cross‐sectional or volume measurements of the TN are not available in horses.

The objective of this study was to determine whether horses with ITMHS exhibit measurable side‐to‐side asymmetry of the TN on high‐field MRI. We therefore hypothesized that the side‐to‐side difference in trigeminal‐nerve cross‐sectional area (TNCSA) would be significantly larger in ITMHS horses than in non‐HS controls, irrespective of whether the size change represents focal atrophy, swelling, or another morphological alteration.

## 2 Materials and Methods

### 2.1 Animals and Inclusion Criteria

The study was designed as a retrospective case–control study. MRI studies of the equine brain performed at the Clinic for Horses (University of Veterinary Medicine Hannover, Foundation, Germany) in the years 2021 and 2022 were included. The included horses were subdivided into two groups (ITMHS and control) according to the final diagnosis. Mandatory information for both groups included age, sex, bodyweight, and breed of each horse. In addition, it was essential to ensure that at least three of the four measurement points (MPs) were visible in the field of view of the MRI scan and that the measurement of the TN was not affected by artifacts. Details regarding the MPs are given at the end of this section.

Criteria for inclusion in the ITMHS group were HS signs reported by the owner and having undergone a comprehensive diagnostic protocol to exclude secondary HS as previously reported in 2022 [3]. All horses with suspected HS were assessed by a veterinarian in‐clinic and via videos provided by the owner, ensuring that the diagnosis of HS signs was not based on owner reports alone. Further examinations included clinical, neurological, and ophthalmologic examination, grading of HS at rest and during exercise, laboratory tests, endoscopy of the upper respiratory tract, computed tomography of the skull, and MRI of the brain and surrounding tissues. All procedures were performed under the supervision of a board‐certified specialist in equine internal medicine. The diagnosis of ITMHS was made when there were no findings that were likely to induce secondary HS. The duration of the signs was recorded using an owner questionnaire and documented precisely to the month.

If a potential secondary cause was initially identified and treated but the HS persisted for ≥ 6 months without improvement, the horse was classified as ITMHS, indicating that the suspected cause was unlikely to be the true underlying etiology.

Horses in the control group were referred for conditions unrelated to HS and underwent brain MRI as part of the diagnostic work‐up for their primary disease. Inclusion criteria for the control horses were (i) history and clinical examination revealed no evidence of HS and (ii) the confirmed underlying disease was considered unlikely to affect trigeminal‐nerve morphology. The animals were cared for in accordance with local laws and good veterinary practice. This study was approved by the Animal Welfare Officer of the University of Veterinary Medicine Hannover, Germany (TiHo_EA_12_17–24). The study was compatible with the animal welfare guidelines of the University of Veterinary Medicine Hannover, the German Animal Welfare Act, and Directive 2010/63/EU of the European Parliament and of the Council of 22 September 2010 on the protection of animals used for scientific purposes. Owner's consent for retrospective use of medical data was obtained.

### 2.2 Image Acquisition

MRI examinations of the brain and the surrounding tissue were performed under general anesthesia after a standardized protocol. The horses were premedicated with acepromazine (0.05 mg/kg IM; Tranquisol, CP‐Pharma Handelsgesellschaft mbH, Burgdorf, Germany), xylazine (0.8 mg/kg IV; Xylavet, CP‐Pharma) and flunixin‐meglumine (1.1 mg/kg IV; Flunidol RPS, CP‐Pharma); general anesthesia was induced with ketamine (2.5 mg/kg IV; Narketan, Vétoquinol GmbH, Ravensburg, Germany) and diazepam (0.05 mg/kg IV; Ziapam, TVM, Lempdes, France). Initially, general anesthesia was maintained by constant rate infusion (ketamine, guaifenesin and xylazine) for approximately 10 min. All horses underwent a computed tomographic evaluation of the skull immediately before the MRI study during the same general anesthesia. During the MRI scan, anesthesia was maintained with isoflurane (Isofluran CP, CP‐Pharma) in 100% oxygen and constant rate infusion of xylazine. Dobutamine (Dobutamine Liquid Fresenius; Fresenius Kabi Deutschland GmbH, Bad Homburg, Germany) was used as required to maintain adequate blood pressure (0.33–0.66 μg/kg/min IV). Horses were placed in dorsal recumbency with the forehead on a torso coil for MRI scans (3‐Tesla, Philips Achieva dStream, Philips, Eindhoven, The Netherlands). The head was positioned so that the brain lay centrally within the 60‐cm coil pad. The MRI scans were evaluated for abnormal findings by an EBVS specialist in veterinary diagnostic imaging. All horses were scanned with a standard MRI protocol including 3‐D T1‐weighted, T2‐weighted turbo spin‐echo, and balanced gradient‐echo sequences. Anesthesia time, monitored values (heart rate, respiratory rate, body temperature, invasively measured arterial blood pressure, oxygenation), mechanical ventilation settings, and drug application rates, important events, and the scanning time were reported. The recovery was slope‐assisted and took place in a padded recovery box.

### 2.3 Definition of Measurement Points (MP)

**FIGURE 1:**
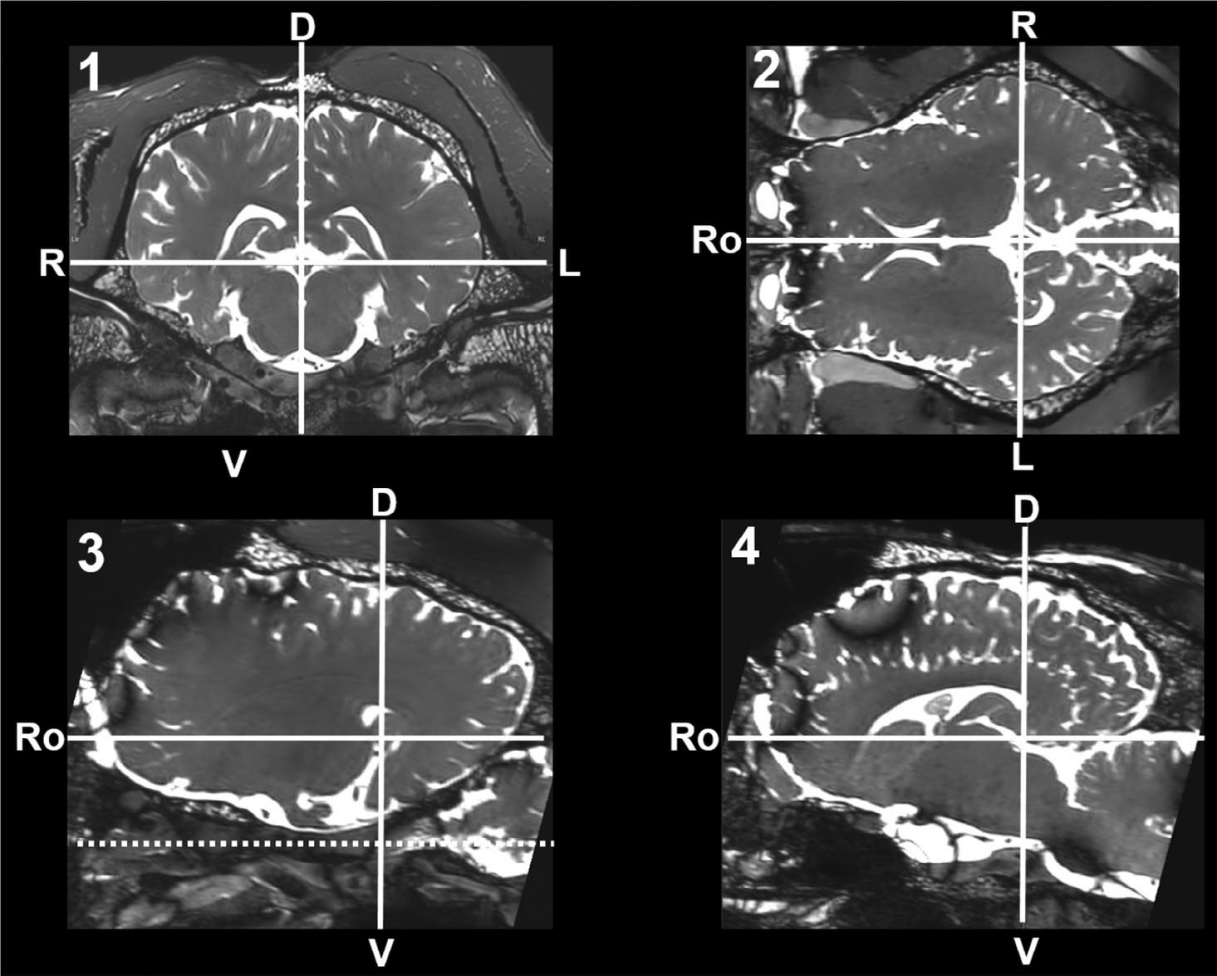
Four images from a balanced gradient echo sequence of the brain of one horse with headshaking: (1) transverse image, (2) dorsal image, (3) parasagittal image, (4) mid‐sagittal image. Bold lines, marker for plane adjustment; D, dorsal; dotted line, course of ophthalmic and maxillary nerve; L, left; *R*, right; Ro, rostral; V, ventral.

**FIGURE 2:**
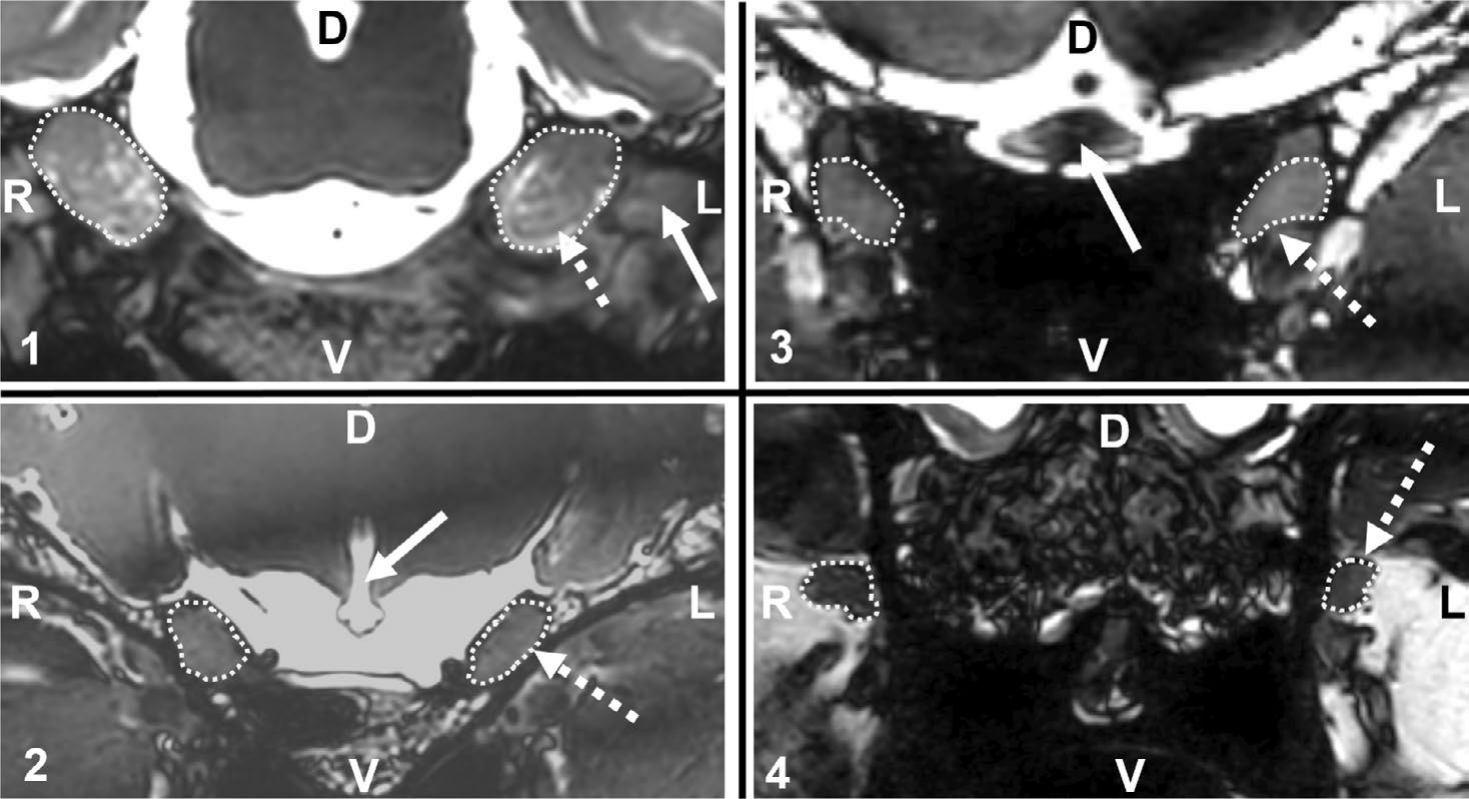
Four images from a balanced gradient echo sequence of the brain of one horse with headshaking. The figure is subdivided into four quadrants: One for each measurement point (1–4). Each quadrant shows a transverse MRI image displaying the trigeminal nerves and surrounding tissue. The dotted circle indicates the trigeminal nerve cross‐sectional area (measurement point). The left trigeminal nerve is indicated by the dotted arrow. Bold arrows indicate anatomical landmarks: (1) mandibular nerve, (2) infundibular recess, (3) optic chiasm, D, dorsal; L, left; *R*, right; V, ventral. Note the slight difference in cross‐sectional area, for example at measurement point 2: right 50 mm^2^, left 61 mm^2^.

The MRI studies of two horses (one from the ITMHS group, one from the control group) were used to define four MPs and to define reproducible measurement conditions. Standardized adjustment of the image planes was defined according to the course of the TN (Figure 1). All measurements of the TNCSA were performed in the transverse plane, orthogonal to the TN. Therefore, the following criteria were defined: the sagittal plane had to be parallel to the median plane of the head; the dorsal plane had to be parallel to the intracranial part of the maxillary and ophthalmic nerve (Figure 1.3, dotted line). Finally, the transverse plane cut both planes perpendicularly. The adjustment of the transverse plane had to fulfill two additional criteria: (1) the nerve root of both TNs needed to be visible on one slice and (2) both frontal poles of the cerebrum had to be visible on the same slice. If necessary, realignment was performed in 3D MRI sequences. The window level was adjusted for optimal visualization of the TN. The TN branches running in the rostro‐caudal direction (ophthalmic and maxillary nerve) were chosen for further measurements. Four MPs of the TNCSA were defined (Figure 2). Therefore, the rostro‐caudal position of the transverse plane was defined by anatomical landmarks. The most caudal MP was just rostral to the region where the mandibular nerve emerges from the trigeminal ganglion, so the measurement included the rostral aspect of the trigeminal ganglion (Figure 2.1). The second MP was defined at the level of the pituitary gland where the infundibular recess leaves the brainstem (Figure 2.2); this MP contained fibers of the ophthalmic and maxillary nerves. MP 3 was set at the level of the optic chiasm (Figure 2.3), and the fourth MP was located rostral to the alar canal and caudal to the pterygopalatine fossa (Figure 2.4), where only maxillary nerve fibers were included.

### 2.4 Image Analysis

Measurements were performed using the DICOM viewer EasyImage (VetZ GmbH, Isernhagen, Germany). The MRI sequence that best visualized the TN was selected for that particular horse, and all subsequent measurements were performed in the same sequence. When available, a 3D T2‐weighted balanced fast field echo sequence was used (TR 7.86 msec., TE 4.43 msec., flip angle 60°, slice thickness 1 mm, receiver bandwidth 433 Hz/pixel, matrix 640 × 640 pixels, FOV 180 mm), and occasionally, a T1‐weighted 3D SENSE was used (TR 7.7 msec., TE 3.6 msec., flip angle 90°, slice thickness 1.1 mm, receiver bandwidth 209 Hz/pixel, matrix 512 × 512 pixels, FOV 280 mm). To eliminate the potential for sequence‐related bias in the TNCSA comparison, measurements were performed on each horse using the same sequence throughout. The TNCSA was measured bilaterally by outlining the outer contour of the nerve using a measurement tool. In the event that an MP was not clearly visible, for example due to artifacts or because the field of view was too small, no measurement was carried out on this MP.

### 2.5 Statistical Analysis

Each measurement was carried out four times with a minimum interval of 3 weeks within repeated measurements; three repetitions were documented with a measurement accuracy of 1 square millimeter to assess measurement accuracy; the fourth repetition was documented with an accuracy of 0.01 square millimeter, as this precise value enabled the use of a linear model. All measurements were carried out by a single examiner (ECEIM resident).

Data analysis was performed with R version 4.3.2. Reliability of measurements was established using the preliminary measurements performed in triplicate at intervals of three or more weeks. Values were rounded to the nearest integer.

Pearson's correlation coefficient was determined and the intraclass correlation coefficient (ICC) calculated from a one‐way model, where subjects are random and raters fixed, as implemented in the “irr” R‐package [21].

Mixed linear models fitted by REML using the “nlme” R‐package [22] were used to investigate the effect of group, MP, and the group × MP interaction on TNCSA, maximal TNCSA, or TNCSA asymmetry within MP.

The models allowed for a group and MP‐specific variance, with Horse as a random effect. Post hoc analyses were conducted with the “emmeans” R‐package [23]. *p*‐values were obtained by Wald tests, and statistical significance was accepted at *p* < 0.05.

Absence of major departures from normality and homoskedasticity was ensured by visual inspection of model residuals (Q–Q plots).

To determine the effects of age and bodyweight, the baseline model (refitted by ML) was compared to models including age or bodyweight with an interaction with MP or a three‐way interaction with MP and group. In both cases, the final model selected by AIC [24] included group, MP, the group × MP interaction, and either age or bodyweight along with interactions between these variables and MP.

Cut‐offs to differentiate horses with ITMHS from controls were obtained for each MP by maximizing the Youden‐Index after kernel smoothing the distributions of the two classes, as implemented in the “cutpointr” R‐package [25]. The performance of the cut‐offs was described based on the same data as used for cut‐off estimation and was therefore expected to overestimate actual performance.

## 3 Results

### 3.1 Animals

A total of 33 equine brain MRI scans were identified; however, only 26 met the inclusion criteria. The study group consisted of different breeds (ITMHS: 17 Warmbloods, two Thoroughbreds, one Quarter Horse//control group: four Warmbloods, one Quarter Horse, one pony), with comparable body weight (ITMHS 540 ± 45.3 kg; control 477 ± 154 kg) and age across groups (ITMHS 8.45 ± 3.28 years; control 10.0 ± 5.14 years). The horses in the control group were diagnosed with cryptogenic epilepsy (*n* = 3), cortical degeneration (*n* = 1), cerebellar disease (*n* = 1), and vestibular disease (*n* = 1) based on clinical examination and imaging findings.

### 3.2 MRI Sequences

Subjective assessment of all available MRI sequences revealed a 3D balanced gradient echo sequence (*n* = 24) or a T1‐weighted 3D SENSE sequence (*n* = 2) as best suitable for the measurement procedure. In the balanced gradient echo sequences, the contrast of the nerve to the surrounding tissue (bone, fluid) was clearest, the resolution was high, and fewer artifacts were present. MP 1 and MP 2 were visible in good quality in all horses (*n* = 26). MP 3 was not measurable in one horse and only partially measurable in seven horses. In these cases, there were band artifacts on MP 3, which made it difficult to clearly differentiate the nerve from the surrounding tissue. In addition, at this MP, the two parts of the TN (ophthalmic nerve and maxillary nerve) were visually separated in 10 horses and still combined in 16 horses. Nevertheless, the anatomy was comparable on both sides, so side‐by‐side evaluation remained feasible. In two of the 26 MRI scans performed, MP 4 was not included in the field of view.

### 3.3 Reliability of Measurements

The overall ICC indicated an excellent measurement reliability (ICC = 0.981, *F*_(201,404)_ = 158, *p* < 0.0001, 95% CI = [0.976–0.985]). Split by location, the reliability of the repeated measurements was good for MP 4 (ICC = 0.817, CI = [0.726–0.886]), and excellent for MP 1 (ICC = 0.981, CI = [0.971–0.989]), MP 2 (ICC = 0.965, CI = [0.946–0.979]), and MP 3 (ICC = 0.946, CI = [0.915–0.967]). The correlation, i.e., the repeatability between repeated measurements was excellent as well (Figure S1).

### 3.4 Absolute TNCSA

**FIGURE 3:**
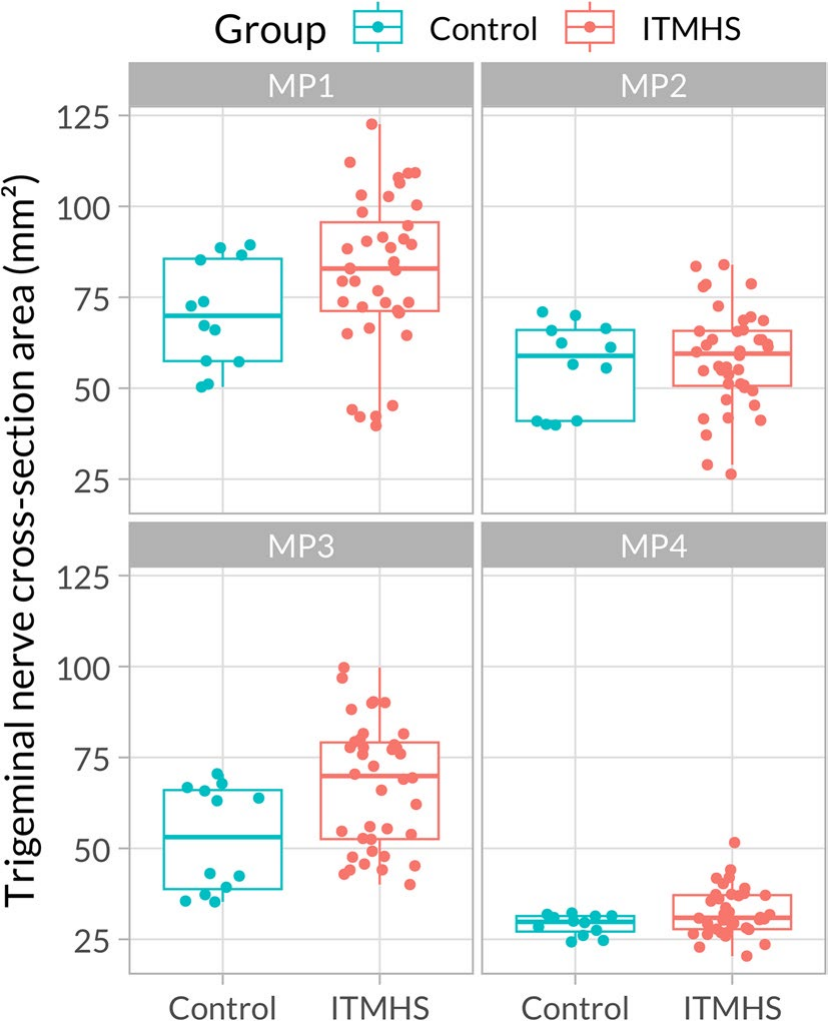
Trigeminal nerve cross‐sectional area (mm^2^) by group (control/Headshaking [ITMHS]) and location (MP 1‐MP 4). Individual values are indicated by dots. MP, measurement point.

The mixed linear model indicated overall differences across locations (*F*_(3,170)_ = 159.026, *p* < 0.001) with significant interactions between group and MP (*F*_(3,170)_ = 6.249, *p* < 0.001). However, the main effect of group (*F*_(1,24)_ = 0.029, *p* = 0.866) was not significant (i.e., the horses with ITMHS did not appear to have larger or smaller nerve cross‐sections independently of MP [Figure 3]). Moreover, there was no significant difference in TNCSA between the groups at any MP (Table 1). While horses with ITMHS did have slightly larger TNCSA, this was not significant (Table 1). TNCSA was largest at MP 1 for both groups (marginal mean [95% CI]: control = 70.5 [59.6–81.4] mm^2^, ITMHS = 81.9 [81.9–88.6] mm^2^) and decreased in the rostral direction to MP 4 (control = 29.0 [18.0–40.0] mm^2^, ITMHS = 33.3 [27.2–39.4] mm^2^), with similar areas at MP 2 (control = 55.9 [46.6–65.3] mm^2^, ITMHS = 58.2 [52.6–63.7] mm^2^), and MP 3 (control = 52.6 [42.5–62.6] mm^2^, ITMHS = 66.9 [59.6–74.1] mm^2^; Figure 3).

**TABLE 1:** Results of pairwise comparisons of the absolute trigeminal nerve cross‐sectional (TNCS) area between control and ITMHS at different locations (MP 1‐MP 4). *p*‐values were adjusted for multiple comparisons with the Dunnett method.

Group comparison	Location	Estimate and 95% CI	SE	DF	*t*‐ratio	*p*
Control vs. ITMHS	MP 1	−11.386 [−27.691–4.919]	6.198	24	−1.837	0.233
Control vs. ITMHS	MP 2	−2.241 [−16.1–11.617]	5.268	24	−0.425	0.953
Control vs. ITMHS	MP 3	−14.298 [−30.148–1.551]	6.025	24	−2.373	0.086
Control vs. ITMHS	MP 4	−4.252 [−20.313–11.808]	6.106	24	−0.696	0.855

Abbreviations: CI, confidence interval; DF, degree of freedom; ITMHS, idiopathic trigeminal‐mediated headshaking; MP, measurement point; SE, standard error.

### 3.5 Effect of Age

The main effect of age (*F*_(1,23)_ = 0.857, *p* = 0.36) was not significant, but a significant interaction between MP and age (*F*_(3,167)_ = 18.2, *p* < 0.001) was apparent. This translated into increases in the TNCSA of 0.623 and 1.823 mm^2^ per year of age at MP 2 and MP 3, respectively (*p* ≤ 0.004), while this effect was not significant at MP 1 and MP 4 (Figure S2). Therefore, the effect of age varied according to location, but horses with ITMHS did not appear to age differently from the controls.

### 3.6 Effect of Bodyweight

**FIGURE 4:**
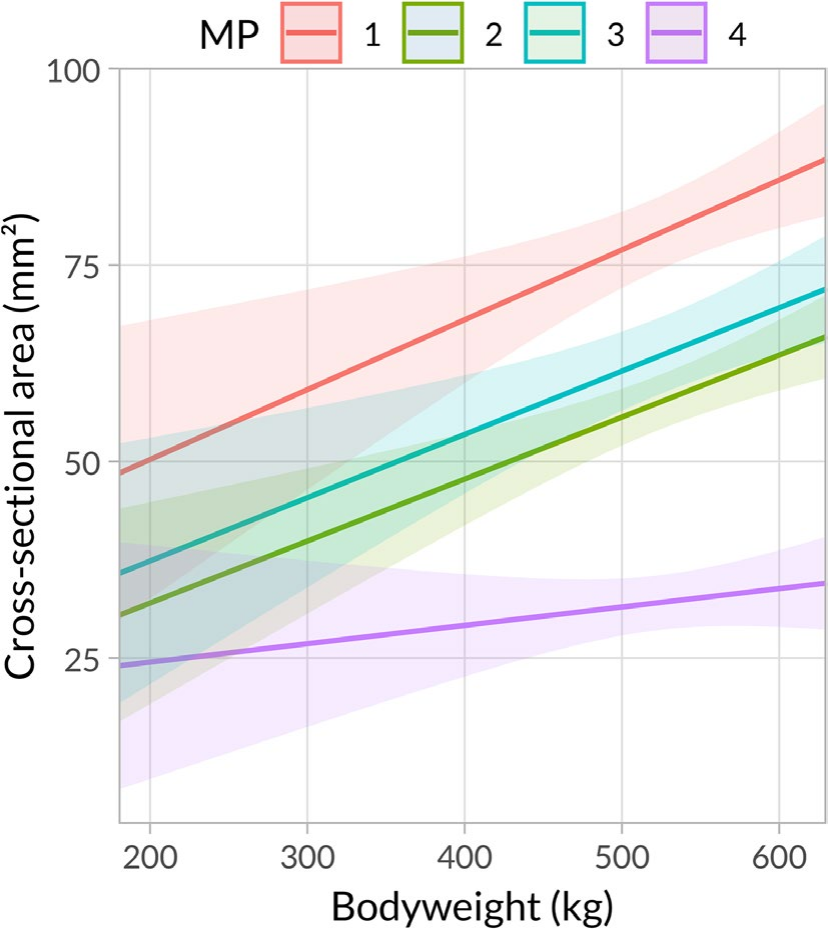
Visualization of the marginal mean trigeminal nerve cross‐sectional area (mm^2^) and 95% confidence interval at four measurement points (MP 1‐MP 4) depending on bodyweight averaged across groups.

Bodyweight had a significant impact on TNCSA both as a main effect (*F*_(1,23)_ = 14.659, *p* = 0.001) and in interaction with MP (*F*_(3,167)_ = 5.838, *p* = 0.001). This effect was positive (Figure 4, Table S1) but not associated with ITMHS (*F*_(1,23)_ = 2.556, *p* = 0.122).

### 3.7 Maximal TNCSA

The mean maximal TNCSA was larger by 15.17 ± 7.05 mm^2^ in the ITMHS than in the control group independently of MP. Despite the significant effect of group (*F*_(1,24)_ = 34.313, *p* < 0.001), there was a marked overlap between the groups, indicating the limited ability of maximal TNCSA to distinguish horses with ITMHS from control horses (Figure S3).

### 3.8 TNCSA Asymmetry

**FIGURE 5:**
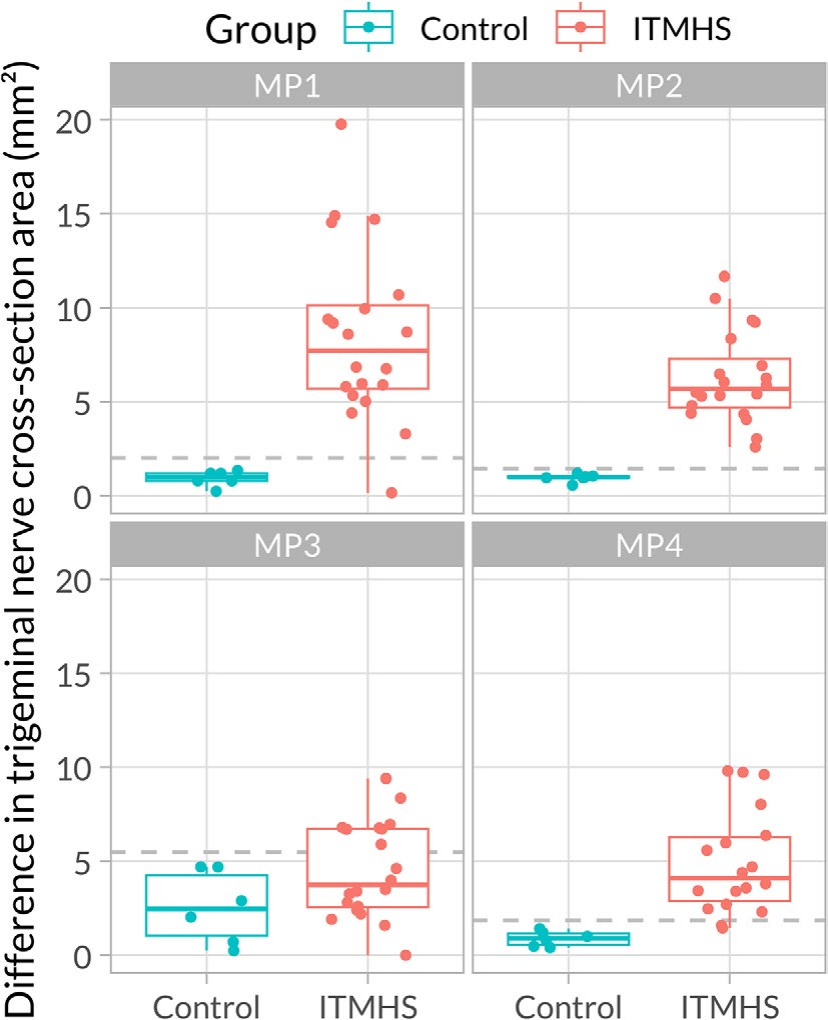
Asymmetry of trigeminal nerve cross‐sectional area in horses with headshaking (ITMHS) compared to the control group grouped by location (measurement points [MP] 1–4). Individual values are indicated by dots.

The TNCSA asymmetry (side‐to‐side difference) ranged from 0 to 5 mm^2^ in the control group and between 0 and 20 mm^2^ in the ITMHS group, with significant differences between the groups as the main effect (*F*_(1,24)_ = 161.036, *p* < 0.001), and in interaction with MP (*F*_(3,70)_ = 5.784, *p* = 0.001) attributable to locations MP 1, MP 2, and MP 4 (Figure 5, Table 2).

**TABLE 2:** Results of location (MP 1‐MP 4) specific comparison of trigeminal nerve cross‐sectional area asymmetry between control and ITMHS. *p*‐values were adjusted for multiple comparisons with the Dunnett method.

Group comparison	Location	Estimate	SE	DF	*t*‐ratio	*p*
Control vs. ITMHS	MP 1	−7.575	1.062	24	−7.132	< 0.001
Control vs. ITMHS	MP 2	−5.320	0.549	24	−9.691	< 0.001
Control vs. ITMHS	MP 3	−1.949	0.993	24	−1.962	0.188
Control vs. ITMHS	MP 4	−4.063	0.668	24	−6.080	< 0.001

Abbreviations: DF, degree of freedom; ITMHS, idiopathic trigeminal‐mediated headshaking; MP, measurement point; SE, standard error.

### 3.9 Predictive Capability

Cut‐offs to differentiate horses with ITMHS from controls based on TNCSA asymmetry were obtained for each MP (Table 3).

**TABLE 3:** Cut‐off values of trigeminal nerve cross‐sectional area asymmetry (mm^2^) for each location (MP 1‐MP 4) and their respective performance on the same dataset.

Location	Cut‐off	Accuracy	Sensitivity	Specificity
MP 1	2.011	0.962	0.950	1
MP 2	1.450	1.000	1.000	1
MP 3	5.483	0.538	0.400	1
MP 4	1.846	0.917	0.889	1

Abbreviation: MP, measurement point.

### 3.10 Effect of Reported Duration of Clinical Signs on TNCSA Asymmetry

There was no significant effect of the reported duration of HS signs on TNCSA side‐to‐side difference (*F*_(1,16)_ = 2.226, *p* = 0.155).

## 4 Discussion

This study reveals an increased TNCSA side‐to‐side difference (TNCSA asymmetry) in horses with ITMHS compared to the control horses. ITMHS is probably related to a TN dysfunction [4, 7, 9], but morphological alterations of the nerve have not been described. No previous studies have shown side‐to‐side differences of the equine TN. Electrophysiological examinations [7, 8] and histopathological examinations [26, 27] have been performed bilaterally in small cohorts, but side‐to‐side comparison was not the main objective of those studies. While electrophysiological studies often show bilateral TN dysfunction in ITMHS [7], our results suggest that morphological changes can be asymmetrically pronounced. This does not exclude bilateral disease; instead, one side might be more severely affected, highlighting the potential heterogeneity of ITMHS. In human medicine, MRI studies revealed TN asymmetry in patients with severe trigeminal neuralgia [14]; a disease suspected to share similarities with ITMHS. The reduced size or volume of the TN is associated with the ipsilateral side of facial pain and is consistent with atrophy of the nerve in humans [14, 15]. The results of the present study indicate TNCSA asymmetry in horses with ITMHS; however, the observed asymmetry should not be interpreted as evidence of a specific pathological process. In the context of findings in human medicine [14, 15], and taking into account a non‐peer‐reviewed study (conference proceedings) that found degenerative changes in the TN in horses with ITMHS [26], atrophy of one TN was considered the most likely cause for the detected TN asymmetry. As the data showed that the TNCSA was slightly larger in ITMHS, swelling (e.g., due to inflammation) of one nerve could also be a possible reason for asymmetry. However, TN inflammation has not been reported in horses with ITMHS. In contrast, available electrophysiological work demonstrates symmetrical latency, amplitude, and conduction velocity; the sole repeatable difference is a reduced activation threshold in affected horses, indicating bilateral hypersensitivity without large‐fiber demyelination [7, 8]. Unfortunately, TN degeneration was not reproducibly detectable in different study groups histologically [7, 27]. The increased TNCSA asymmetry in the ITMHS group suggested more pronounced unilateral disease processes. This is supported by the fact that TNCSA asymmetry in the control group was low, indicating symmetry as the typical situation, as expected from human medicine [28]. Therefore, asymmetry is a promising indicator of ITMHS in this study sample. Assuming that ITMHS is a predominantly unilateral disease, future treatment might be unilateral.

Beyond that, not all horses in the ITMHS group had marked TN asymmetry. While TNCSA asymmetry was measurable at multiple MPs—particularly MP1, MP2, and MP4—no distinct focal abnormalities of the TN were observed on MRI. It remains unclear whether the observed differences in nerve diameter reflect primary changes of the nerve fibers themselves or secondary alterations in the surrounding perineural tissue. These findings could be due to different subtypes of ITMHS (some with marked asymmetry and others without) or different disease stages (asymmetry develops over the course of the disease, or initial swelling is followed by atrophy). Given the variability in the subarachnoid space extent along the branches of the TN [20], this must also be considered as a potential factor influencing the results, especially at MP1. However, without histological analysis, this could not be investigated retrospectively.

The cut‐off values for discriminating ITMHS from controls performed well in the study group but need to be validated in additional clinical patients to obtain reliable performance estimates. Moreover, the natural variation of TNCSA in healthy horses could be larger than estimated on this relatively small sample size.

This study focused on the parts of the TN that were easy to identify according to results of previous studies [16, 17, 18] and are likely to be clinically relevant for ITMHS. Specifically, the ophthalmic and maxillary branches of the TN were evaluated together along their shared course within the cranial cavity at the more caudal MPs (MP 1, MP 2, and MP 3). At MP 4, located outside the cranial cavity, only the maxillary nerve was assessed. Since the ophthalmic and maxillary nerves could not be differentiated within the cranial cavity, both nerves were measured together. The maxillary nerve was chosen for measurements outside of the cranial cavity. It has been shown to be affected by functional disorders in ITMHS [7] and is targeted during percutaneous electrical nerve stimulation that can improve clinical signs [3, 10, 29].

This study could not demonstrate an effect of the duration of ITMHS on the TNCSA asymmetry. Duration data relied on owner‐reported information and could not be independently verified. Furthermore, the severity of clinical signs and the rate of clinical progression might vary among affected horses. This represents a limitation of the study.

The absolute TNCSA did not differ significantly between groups. Bodyweight influenced TNCSA, with heavier horses having greater TNCSA. This finding was expected because heavier (and presumably taller) horses have larger heads with thicker nerves. On the other hand, the effect of bodyweight did not differ significantly between the ITMHS and the control groups and was therefore not included in further analyses.

An age‐related effect on TNCSA was detectable at certain MPs. Nevertheless, as there was no evidence of an influence of ITMHS on these age‐related differences, we assume a symmetrical influence of age on TNCSA. This supports the advantage of using TNCSA asymmetry, which is not influenced by age. Age was therefore not included in further calculations.

When the different MPs were compared, MP 2 was superior in the discrimination of ITMHS and control horses. The asymmetry at this location was smallest in the controls but pronounced in ITMHS horses. Thus, this is the most promising MP for clinical use.

The high ICC and correlation coefficients indicate excellent reliability of the measurement procedure. This supports an adequate definition of MPs, reproducible methodology, and offers the prospect of clinical use. Repeatability of measurements by other examiners and clinical applicability still remain to be evaluated.

The TN was easy to identify in all MRI studies used, which was to be expected based on previous studies [16, 17, 18]. Balanced gradient echo sequences proved superior for measuring the TN adjacent to the skull base, due to a high contrast with the cerebrospinal fluid, blood vessels, and other surrounding tissues [30]. MP 1, MP 2, and MP 4 are reliably detectable and measurable. MP 3 is more prone to band or susceptibility artifacts due to its location in the alar canal (surrounded by bone and adjacent to air‐filled structures). Balanced gradient echo sequences have a good contrast between the target tissue and the surrounding tissue, but band artifacts occur due to local disturbances in the magnetic field, for which this sequence is more susceptible than others [31]. Balanced gradient echo sequences are also comparatively rapid, which shortens scanning time and helps minimize overall anesthetic duration in clinical settings. If an MP was compromised by artifacts, no measurement was performed. In addition, the degree of separation of the ophthalmic nerve and the maxillary nerve differed between individuals but not within individuals at MP 3. Therefore, it is recommended to use the regions of MP 1, MP 2, and MP 4 as points of interest for further measurements, preferably MP 2, as mentioned above.

Due to its retrospective design, this study has some limitations. First, the study is limited by the modest number of control horses and by breed and body size disparity between groups; absolute nerve caliber therefore has wide confidence intervals, and our findings must be validated in a larger, weight‐matched cohort. This potential bias was mitigated by using intra‐individual side‐to‐side comparison of TNCSA. This approach was supported by the reported TN symmetry in humans [28]. TN symmetry was confirmed in the control group of this study. Therefore, the variable “TN asymmetry” is independent of body weight or age of the horses. Secondly, the control group included horses with intracranial pathology, although only horses with diseases unlikely to affect the TN morphology were selected. The diseases in the control horses were located in the cerebellum, cerebrum, or vestibular system. A detailed review of the medical records and an owner interview were used to rule out any HS signs in these horses.

Furthermore, histopathological examination of the TN was not performed. This limits the study to a descriptive approach and precludes the identification of pathophysiological mechanisms. Finally, the fact that all measurements were performed by one non‐blinded examiner limits generalizability to other examiners and reduces the clinical applicability. This limitation should be outweighed by having used repeated measurements, as mentioned above.

In conclusion, this study describes a bilateral asymmetry of the TN occurring in horses with ITMHS. Unilaterally enhanced changes highlight the role of the TN in ITMHS. MP 1, 2, and 4 appeared to be suitable for detecting ITMHS‐related TNCSA asymmetry and are recommended for clinical use. The results indicate unilateral TN disease in ITMHS, which potentially has implications for future treatment.

## Disclosure

Authors declare no off‐label use of antimicrobials.

## Ethics Statement

This study was approved by the Animal Welfare Officer of the University of Veterinary Medicine Hannover, Germany (TiHo_EA_12_17‐24). The study was compatible with the animal welfare guidelines of the University of Veterinary Medicine Hannover, the German Animal Welfare Act, and Directive 2010/63/EU of the European Parliament and of the Council of September 22, 2010 on the protection of animals used for scientific purposes. All horse owners agreed to the storage and processing of data for research purposes. Authors declare human ethics approval was not needed.

## Conflicts of Interest

The authors declare no conflicts of interest.

## Supporting information


**Data S1:** Supporting Information.

**Figure S1:** Correlation matrix for all three repeated measurements of the trigeminal nerve cross‐sectional area by replicates (R1, R2, R3). Pearson's correlation coefficient (p) is given in the upper matrix triangle.

**Figure S2:** Visualization of the marginal mean trigeminal nerve cross‐sectional area (mm^2^) and 95% confidence interval at four measurement points (MP 1‐MP 4) depending on bodyweight averaged across groups. Individual measurements are indicated by dots.

**Figure S3:** Maximal trigeminal nerve cross‐sectional area (mm^2^) by group (control/headshaking [ITMHS]) and location (MP 1‐MP 4). Individual values are indicated by dots. MP, measurement point.

**Table S1:** Values by which the cross‐sectional area increase at each location for an increase in bodyweight of 100 kg. Prediction was obtained for 500–600 kg bwt, and the confidence intervals are specific to the bodyweight range.
