# Supplementary material for: Trigeminal Nerve Asymmetry in Horses With Idiopathic Trigeminal‐Mediated Headshaking: A Retrospective Case‐Control Magnetic Resonance Imaging Study
Source: J Vet Intern Med. 2025 Jul 31;39(5):e70196. doi: 10.1111/jvim.70196 (PMC12311309; doi:10.1111/jvim.70196)
Supplement: Supplementary file 1 — Data S1: Supporting Information. [file JVIM-39-e70196-s002.pdf]

Table. Raw data of trigeminal nerve cross-sectional area (TNCSA) measurements in 26 horses. Measurements were taken at four predefined measurement points (MP1 - MP4) on the trigeminal nerve, with four repeated measurements per MP on both the left (L) and right (R) side. Each horse was assigned to either the control group or the ITMHS group.

NA = measurement not possible; R = right; L = left; ID = number of horse; MP = measurement point; ITMHS = idiopathic trigeminal-mediated headshaking.

ID	MP_1_R	MP_1_L	MP_2_R	MP_2_L	MP_3_R	MP_3_L	MP_4_R	MP_4_L	Group
1	106	92	79	71	81	78	32	30	ITMHS
2	86	80	54	54	54	57	29	32	ITMHS
3	75	55	47	38	77	69	34	26	ITMHS
4	77	79	49	64	60	64	34	40	ITMHS
5	38	38	35	33	41	37	24	25	ITMHS
6	85	88	67	73	82	83	35	40	ITMHS
7	73	76	50	55	77	75	28	30	ITMHS
8	82	73	61	56	79	76	NA	NA	ITMHS
9	92	83	80	72	76	70	35	26	ITMHS
10	88	92	64	56	80	74	41	37	ITMHS
11	69	62	49	58	NA	NA	24	28	ITMHS
12	65	69	50	56	85	90	48	52	ITMHS
13	107	104	66	56	69	64	47	42	ITMHS
14	74	84	55	64	61	64	37	38	ITMHS
15	99	108	70	64	65	68	NA	NA	ITMHS
16	104	112	53	60	57	52	43	49	ITMHS
17	98	111	84	88	81	75	41	44	ITMHS
18	41	44	30	33	50	42	34	32	ITMHS
19	76	63	72	67	50	46	49	40	ITMHS
20	70	79	70	65	50	54	28	28	ITMHS
21	91	90	66	67	66	65	25	25	Control
22	55	54	40	41	35	36	27	27	Control
23	84	88	51	53	44	40	22	22	Control
24	87	88	62	61	64	62	28	30	Control
25	78	80	58	58	73	71	30	31	Control
26	49	49	39	40	34	35	23	21	Control
1	107	92	78	73	95	89	31	29	ITMHS
2	91	86	63	62	52	58	30	33	ITMHS
3	66	46	50	42	82	78	33	27	ITMHS
4	74	78	49	63	54	57	34	39	ITMHS
5	39	38	42	38	39	38	21	25	ITMHS
6	84	88	76	81	80	83	35	40	ITMHS
7	77	81	56	63	86	87	25	27	ITMHS
8	85	75	61	54	74	80	NA	NA	ITMHS
9	92	85	80	74	78	74	33	22	ITMHS
10	87	93	64	57	85	80	32	30	ITMHS
11	69	60	48	53	NA	NA	24	28	ITMHS
12	67	69	49	55	77	81	26	32	ITMHS
13	105	100	65	54	70	69	45	40	ITMHS

ID	MP_1_R	MP_1_L	MP_2_R	MP_2_L	MP_3_R	MP_3_L	MP_4_R	MP_4_L	Group
14	72	86	46	52	52	54	32	35	ITMHS
15	97	112	67	63	72	79	NA	NA	ITMHS
16	99	110	56	59	44	41	41	51	ITMHS
17	109	125	77	82	80	77	32	37	ITMHS
18	41	44	28	31	46	42	35	32	ITMHS
19	88	72	66	62	53	48	45	39	ITMHS
20	71	78	70	66	52	59	29	30	ITMHS
21	82	82	64	65	61	63	24	23	Control
22	48	47	41	41	32	33	26	27	Control
23	83	86	49	49	43	41	23	23	Control
24	80	80	61	61	65	60	33	35	Control
25	73	74	59	58	54	53	24	25	Control
26	48	48	39	41	36	39	22	20	Control
1	107	92	79	73	91	85	32	30	ITMHS
2	90	85	61	60	56	59	31	34	ITMHS
3	73	53	47	38	75	70	36	28	ITMHS
4	75	79	49	64	56	59	32	37	ITMHS
5	38	38	40	38	39	37	22	26	ITMHS
6	84	88	72	77	80	82	34	39	ITMHS
7	76	80	46	53	85	87	30	32	ITMHS
8	84	74	61	54	73	79	NA	NA	ITMHS
9	92	84	80	73	76	71	32	21	ITMHS
10	88	94	64	56	83	78	32	31	ITMHS
11	70	61	49	58	NA	NA	22	26	ITMHS
12	66	70	50	56	80	84	30	35	ITMHS
13	107	104	66	55	69	66	44	39	ITMHS
14	73	84	51	57	54	56	33	36	ITMHS
15	98	108	68	63	69	76	NA	NA	ITMHS
16	103	112	56	60	48	45	40	50	ITMHS
17	108	121	79	83	79	74	35	40	ITMHS
18	41	44	28	31	37	37	35	33	ITMHS
19	87	71	67	63	51	46	46	38	ITMHS
20	70	79	70	65	50	57	29	29	ITMHS
21	85	85	66	67	62	63	28	28	Control
22	50	51	43	44	34	35	30	31	Control
23	84	88	52	54	43	40	23	23	Control
24	82	82	60	60	64	62	32	34	Control
25	76	78	59	60	60	58	28	29	Control
26	48	48	40	41	36	38	26	24	Control
1	109.24	94.7	68.58	62.1	81.49	88.21	27.81	26.36	ITMHS
2	91.52	84.76	59.02	55.99	47.6	44.1	26.84	30.42	ITMHS
3	64.98	45.21	51.21	41.86	75.98	69.02	36.16	28.13	ITMHS
4	73.52	79.42	50.18	61.85	52.73	55.99	31.78	37.36	ITMHS
5	42.29	42.13	41.18	37.12	44.05	40.06	20.39	22.86	ITMHS
6	84.51	89.53	72.56	77.9	75.83	77.74	30.82	35.52	ITMHS
7	70.81	76.78	46.83	53.76	77.74	79.33	23.58	25.89	ITMHS

ID	MP_1_R	MP_1_L	MP_2_R	MP_2_L	MP_3_R	MP_3_L	MP_4_R	MP_4_L	Group
8	91.04	82.44	66.03	59.98	78.5	72.6	NA	NA	ITMHS
9	100.36	90.4	83.95	78.53	89.92	81.56	39.03	29.31	ITMHS
10	82.99	88.33	63.32	54.96	96.85	90.08	27.72	37.32	ITMHS
11	71.37	64.52	41.58	50.82	NA	NA	26.84	30.27	ITMHS
12	66.51	72.32	45.32	51.21	90.3	99.7	26.52	32.5	ITMHS
13	106.4	103.1	65.6	55.1	69.4	66	44.1	40.3	ITMHS
14	73.6	82.8	49.3	54.8	52.5	54.7	33.7	37.1	ITMHS
15	98.4	109.1	68.7	63.4	70.4	77.2	NA	NA	ITMHS
16	102.7	112.1	55.8	60.2	45.7	42.9	41.8	51.6	ITMHS
17	107.9	122.6	78.7	83.5	80	77.6	37.6	42	ITMHS
18	39.69	44.1	26.36	28.96	47.8	45.2	31.94	29.23	ITMHS
19	88.65	73.76	65.68	61.33	49.22	53.84	36.99	30.61	ITMHS
20	70.69	79.41	69.59	63.33	55.4	62.1	29.43	31.01	ITMHS
21	88.61	89.4	70.01	70.97	66.74	63.84	32.24	31.46	Control
22	57.51	57.27	39.89	40.94	35.52	35.28	26.07	27.48	Control
23	85.26	86.6	56.56	55.55	42.4	43.1	30.99	29.99	Control
24	66.03	67.22	66.43	65.87	67.8	63.1	31.38	31.86	Control
25	72.6	73.8	61.23	62.43	70.5	65.8	28.4	29.6	Control
26	51.13	50.34	40.06	41.02	37.28	39.31	24.69	24.29	Control
