# Supplementary material for: Trigeminal Nerve Asymmetry in Horses With Idiopathic Trigeminal‐Mediated Headshaking: A Retrospective Case‐Control Magnetic Resonance Imaging Study
Source: J Vet Intern Med. 2025 Jul 31;39(5):e70196. doi: 10.1111/jvim.70196 (PMC12311309; doi:10.1111/jvim.70196)
Supplement: Supplementary file 2 — Figure S1: Correlation matrix for all three repeated measurements of the trigeminal nerve cross‐sectional area by replicates (R1, R2, R3). Pearson's correlation coefficient (p) is given in the upper matrix triangle. [file JVIM-39-e70196-s003.pdf]

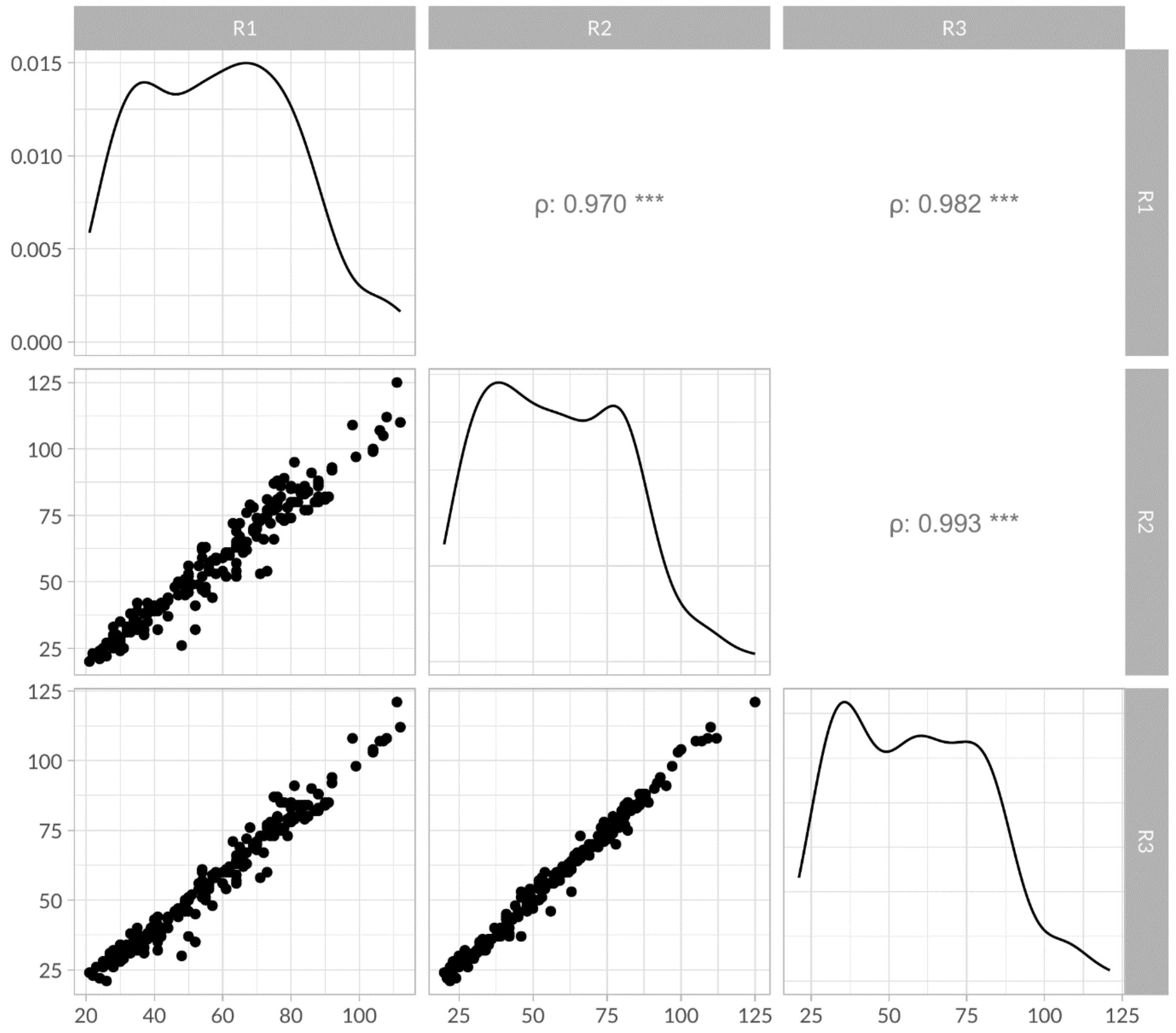

Figure S1: Correlation matrix for all three repeated measurements of the trigeminal nerve cross-sectional area by replicates (R1, R2, R3). Pearson's correlation coefficient (p) is given in the upper matrix triangle.
