# Supplementary material for: Trigeminal Nerve Asymmetry in Horses With Idiopathic Trigeminal‐Mediated Headshaking: A Retrospective Case‐Control Magnetic Resonance Imaging Study
Source: J Vet Intern Med. 2025 Jul 31;39(5):e70196. doi: 10.1111/jvim.70196 (PMC12311309; doi:10.1111/jvim.70196)
Supplement: Supplementary file 3 — Figure S2: Visualization of the marginal mean trigeminal nerve cross‐sectional area (mm2) and 95% confidence interval at four measurement points (MP 1‐MP 4) depending on bodyweight averaged across groups. Individual measurements are indicated by dots. [file JVIM-39-e70196-s005.pdf]

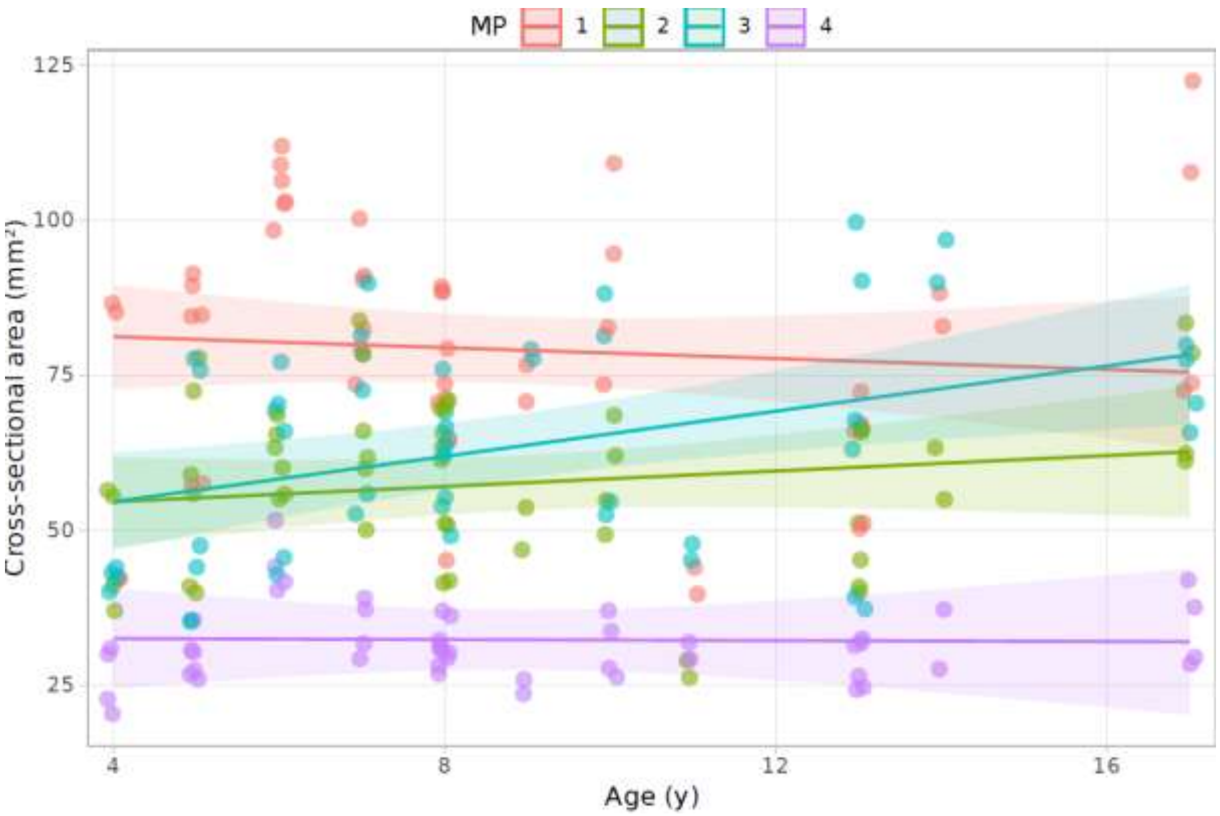

Figure S2: Visualization of the marginal mean trigeminal nerve cross-sectional area (mm2) and 95% confidence interval at four measurement points (MP 1-MP 4) depending on bodyweight averaged across groups. Individual measurements are indicated by dots.
