# Supplementary material for: Trigeminal Nerve Asymmetry in Horses With Idiopathic Trigeminal‐Mediated Headshaking: A Retrospective Case‐Control Magnetic Resonance Imaging Study
Source: J Vet Intern Med. 2025 Jul 31;39(5):e70196. doi: 10.1111/jvim.70196 (PMC12311309; doi:10.1111/jvim.70196)
Supplement: Supplementary file 4 — Figure S3: Maximal trigeminal nerve cross‐sectional area (mm2) by group (control/headshaking [ITMHS]) and location (MP 1‐MP 4). Individual values are indicated by dots. MP, measurement point. [file JVIM-39-e70196-s004.pdf]

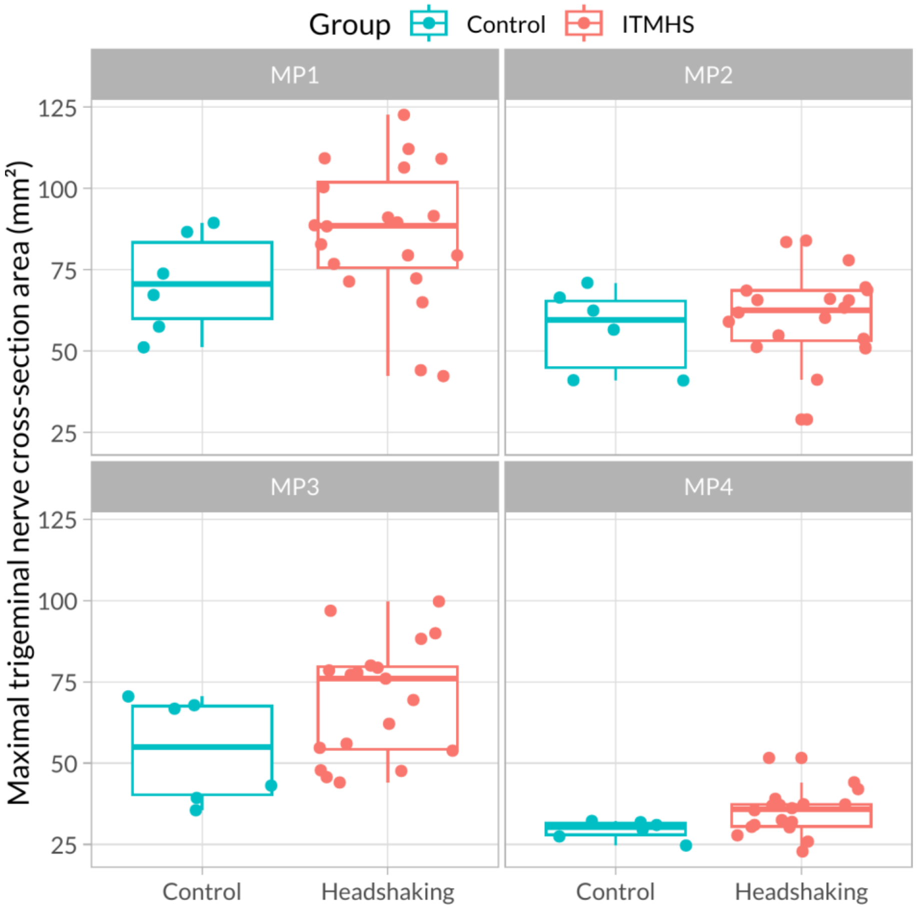

Figure S3: Maximal trigeminal nerve cross-sectional area (mm^2) by group (control / headshaking [ITMHS]) and location (MP 1-MP 4). Individual values are indicated by dots. MP = measurement point.
