# Supplementary material for: Trigeminal Nerve Asymmetry in Horses With Idiopathic Trigeminal‐Mediated Headshaking: A Retrospective Case‐Control Magnetic Resonance Imaging Study
Source: J Vet Intern Med. 2025 Jul 31;39(5):e70196. doi: 10.1111/jvim.70196 (PMC12311309; doi:10.1111/jvim.70196)
Supplement: Supplementary file 5 — Table S1: Values by which the cross‐sectional area increase at each location for an increase in bodyweight of 100 kg. Prediction was obtained for 500–600 kg bwt, and the confidence intervals are specific to the bodyweight range. [file JVIM-39-e70196-s001.pdf]

Table S1: Values by which the cross-sectional area increase at each location for an increase in bodyweight of 100 kg. Prediction was obtained for 500-600 kg bwt, and the confidence intervals are specific to the bodyweight range.

**Location**	**Estimate and 95% CI**	**SE**	**DF**	***t*-ratio**	**p-value**
MP 1	8.894 [3.373-14.416]	2.669	23	3.332	.003
MP 2	7.888 [3.9-11.877]	1.928	23	4.091	< .001
MP 3	8.056 [3.256-12.855]	2.32	23	3.472	.002
MP 4	2.339 [-2.354-7.032]	2.269	23	1.031	.31

Table legend: MP = measurement point; SE = standard error; DF = degree of freedom; CI = confidence interval
